# Karyoevolution of *Crenicichla* heckel 1840 (Cichlidae, Perciformes): a process mediated by inversions

**DOI:** 10.1242/bio.041699

**Published:** 2019-04-29

**Authors:** Luan Felipe da Silva Frade, Bruno Rafael Ribeiro de Almeida, Susana Suely Rodrigues Milhomem-Paixão, Jonathan Stuart Ready, Cleusa Yoshiko Nagamachi, Julio Cesar Pieczarka, Renata Coelho Rodrigues Noronha

**Affiliations:** 1 Centro de Estudos Avançados da Biodiversidade, Universidade Federal do Pará, Campus Guamá, Rua Augusto Corrêa, n° 01. Guamá, Belém, Pará, Brasil; 2 Instituto Federal de Educação, Ciência e Tecnologia de Goiás, campus Valparaıso de Goiás, BR-040, km 6, Avenida Saia Velha, S/N, Área 8, Parque Esplanada V. 72.876-601, Valparaíso de Goiás, Goiás, Brasil

**Keywords:** Neotropical fish, FISH, Karyotypic variation, Ancestral karyotype, Repetitive DNA

## Abstract

*Crenicichla* (Cichliformes, Cichlidae) present a highly conserved diploid number 2n=48 with fundamental numbers varying between 52 and 62. We analyzed four species in order to investigate the role of repetitive DNA in chromosome evolution in the genus. *Crenicichla johanna*, *Crenicichla* cf. *saxatilis* and *Crenicichla* cf. *regani* have 2n=48 (8 m/sm and 40st/a) and FN=56, while *Crenicichla* sp. ‘Xingu I’ has 2n=48 (48 st/a) and FN=48. Different patterns of constitutive heterochromatin distribution were observed including pericentric, interstitial and whole arm C bands. A single chromosome bears 18S rDNA clusters in most species, except *C. johanna*, where population variation exists in terms of the quantity and distribution of clusters and their association with interstitial telomeric sequences. All species showed hybridization of 5S rDNA sequences in an interstitial region on an acrocentric chromosome pair. The karyotypic differences and maintenance of the diploid number supports chromosome evolution mediated by inversions in *Crenicichla*. The telomeric and 18S rDNA sequence association in various chromosomes of *C. johanna* are proposed to represent hotspots for breakage, favoring intra-chromosomal rearrangements. The results suggest that repetitive sequences can contribute to microstructural cytogenetic diversity in *Crenicichla*.

## Abstract (precis)

**Summary:** This paper has a great importance for understanding karyotype evolutionary dynamics in neotropical freshwater fish, focusing on repetitive DNA and the role of inversions in *Crenicichla*.

## INTRODUCTION

*Crenicichla* Heckel, 1840 (Cichliformes) is considered the most species rich genus of Neotropical cichlids with approximately 90 valid species, subdivided in nine groups based on morphology (Kullander, 2003; Varella and Ito, 2017). The genus is widely distributed in all river basins east of the Andes including coastal drainages from Venezuela and the Guianas as far South as the Plata river in Argentina (Kullander, 2003; Kullander et al., 2010; Casciotta et al., 2010; Mattos et al., 2014).

Currently, only 24 species of *Crenicichla* have been analyzed by classical cytogenetic methods. All species have presented a diploid number of 2n=48, an absence of heteromorphic sex chromosomes and an active AgNOR on a single chromosome pair (Benzaquem et al., 2008). Despite the conserved diploid number, members of this genus have shown high interspecific variation in the fundamental number (FN), from FN=52 in *Crenicichla cf. saxatilis* to FN=62 in *Crenicichla niederleinii* (Arai, 2010), suggesting the occurrence of intra-chromosomal rearrangements, including many pericentric inversions (Feldberg et al., 2003) (Table 1).

**Table 1.**
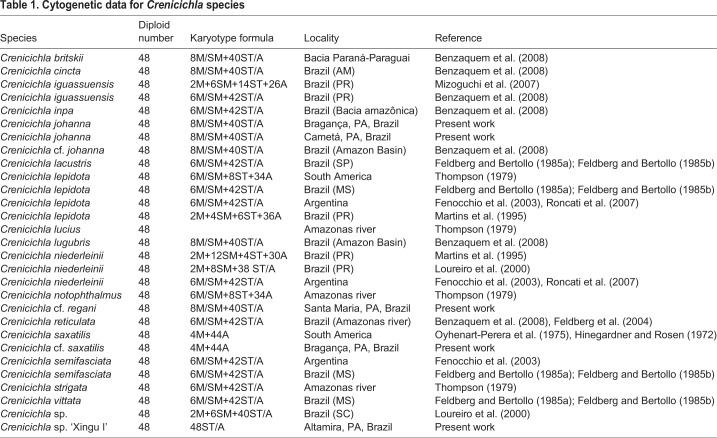
**Cytogenetic data for *Crenicichla* species**

Repetitive DNA constitutes a large part of the genome of eukaryotes, and may be arranged in tandem (satellite DNA, histone genes, etc.) or be dispersed along chromosomes (transposable elements) (Charlesworth et al., 1994). Repetitive sequences may be directly related to karyotypic diversification mechanisms in taxa with stable diploid numbers such as in *Crenicichla*. The presence of repetitive DNA clusters in some genomic regions may represent fragile breakage sites that are repeatedly associated with rearrangements during chromosome evolution (Feschotte and Pritham, 2007; Schneider et al., 2013). In the fish family Loricariidae, the association of 5S rDNA and interstitial telomeric sequences (ITSs) has been proposed to produce hot spots for genomic re-patterning (Barros et al., 2017). In *Crenicichla* chromosomal mapping using repetitive DNA has only been performed in *Crenicichla lepidota* (18S and 5S rDNA), making it impossible to infer the role of these sequences in the karyoevolution of the genus (Perazzo et al., 2011).

As previous cytogenetic characterization of *Crenicichla*, limited almost entirely to classical methods, has not been able to describe the mechanism(s) that result in variation in FN, it is important to investigate the potential role of repetitive DNA in the karyoevolution of the genus. As such, the present study reports cytogenetic data, including mapping of repetitive DNA sequences, for four Amazonian species of *Crenicichla*, considerably increasing coverage for this data in the genus. This data could then be used to analyze genomic organization and investigate the contribution of these repetitive sequences to chromosome evolution in the genus.

## RESULTS

**Fig. 1.**
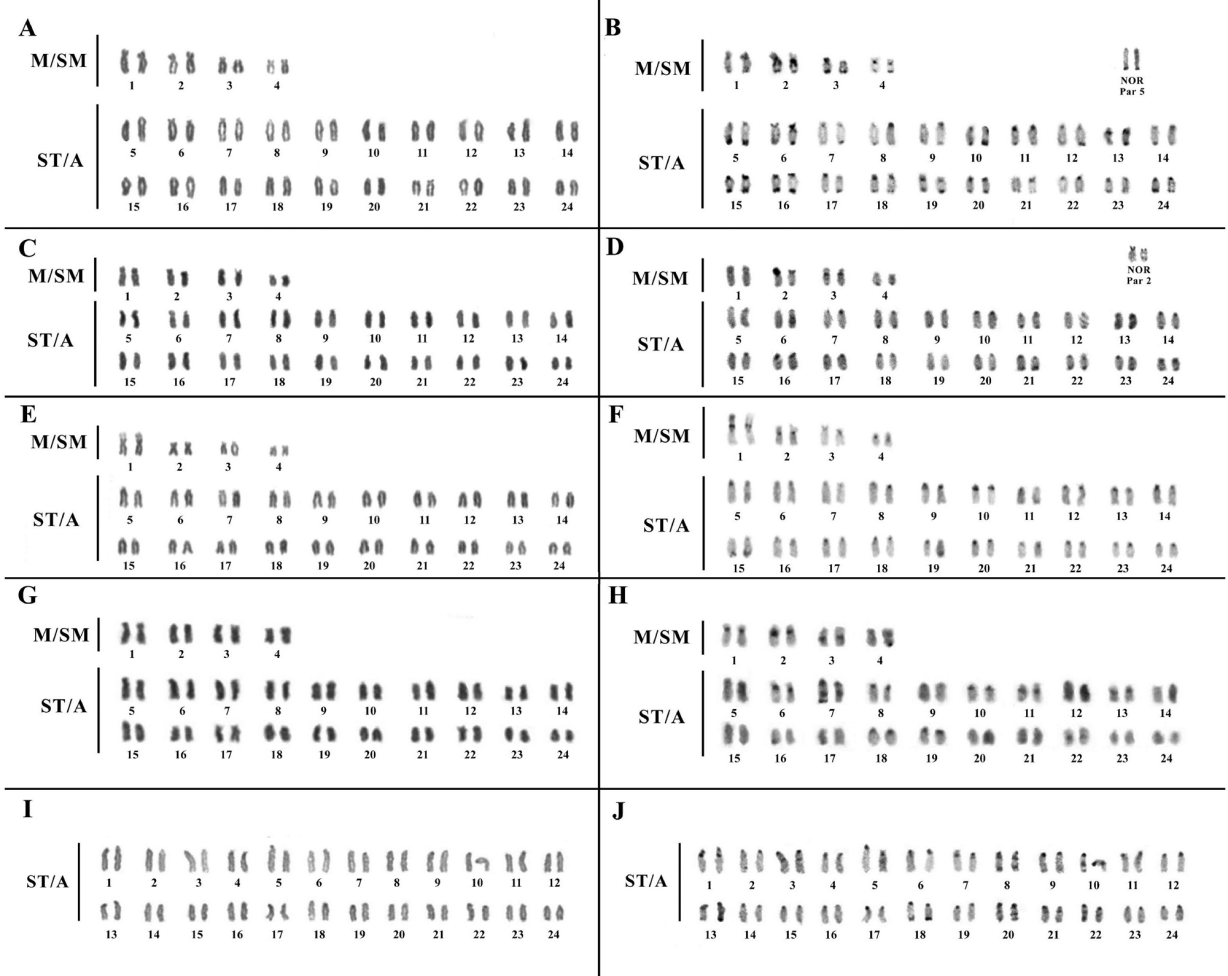
**Karyotypes of *Crenicichla* species based on Giemsa staining (A,C,E,G,I) and C banding (B,D,F,H,J): (A,B) *C. johanna* AB; (C,D) *C. johanna* CA; (E,F) *C.* cf. *saxatilis*; (G,H) *C.* cf. *regani*; (I,J) *C.* sp. ‘Xingu I’.**

All analyzed *Crenicichla* species have 2n=48 and heteromorphic sex chromosomes are absent (Fig. 1). The species *Crenicichla johanna*, *C.* cf*. saxatilis* and *Crenicichla* cf*. regani* present FN=56 and a karyotypic formula of 8 M/SM and 40ST/A (Fig. 1A,C,E,G). In contrast, *Crenicichla* sp. ‘Xingu I’ presented FN=48 and a karyotypic formula of 48ST/A (Fig. 1I,J).

In all four species C banding revealed constitutive heterochromatin (CH) in the pericentromeric region of the majority of chromosomes (Fig. 1B,D,F,H,J). In *C. johanna* some chromosomes presented additional CH in the terminal region (Fig. 1B,D); samples of this species from the population from Cametá (CA) also presented a completely heterochromatic short arm on chromosome pair 2, with size heteromorphism between homologs (Fig. 1D). These characteristics were not found in the population from Abaetetuba (AB) where the heterochromatic block is localized pericentrically and extends only halfway along the long arm (Fig. 1B). In *C.* cf*. saxatilis* additional C bands were observed at a secondary constriction in chromosome pair 1 (Fig. 1F). In *C.* cf*. regani* additional C bands were observed on pairs 3, 7 and 22 (Fig. 1H). In *C.* sp. ‘Xingu I’ interstitial C bands were present on chromosome pairs 1, 3, 7, 8, 10, 20, 21 and 22 (Fig. 1J).

**Fig. 2.**
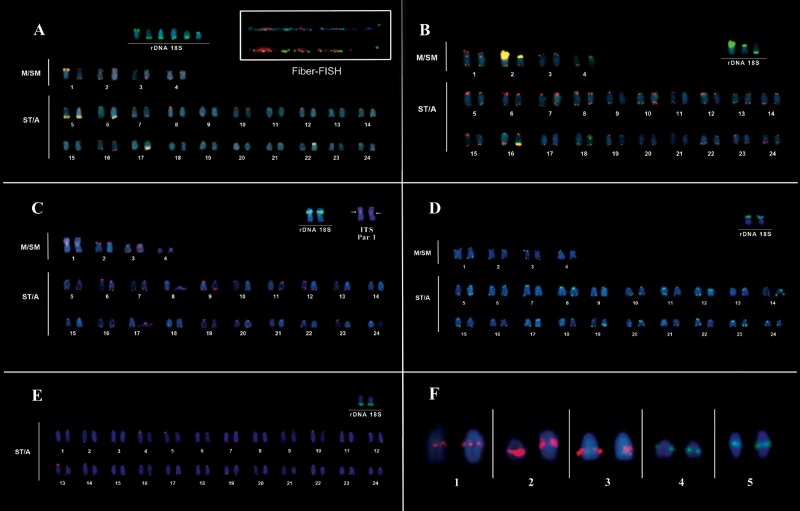
**FISH and Fiber-FISH showing telomeric (red) and 18S rDNA (green) probe hybridization locations in *Crenicichla* species.** (A) *C. johanna* AB, inset: Fiber-FISH and chromosomes bearing 18S rDNA; (B) *C. johanna* CA, inset: chromosomes bearing 18S rDNA; (C) *C.* cf*. saxatilis*, inset: chromosome pair 1 bearing 18S rDNA and pericentromeric ITS; (D) *C.* cf*. regani*, inset: chromosome pair bearing 18S rDNA; (E) *C.* sp. ‘Xingu I’, inset: chromosome pair bearing 18S rDNA. Yellow regions in A and B represent sites with syntenic localization of 18S rDNA and telomeric probes in *C. johanna*. FISH showing 5S rDNA probe hybridization locations in *Crenicichla* species: (F1) *C. johanna* AB; (F2) *C. johanna* CA; (F3) *C.* cf*. saxatilis*; (F4) *C.* cf*. regani*; (F5) *C.* sp. ‘Xingu I’.

Staining with AgNOR showed that in all species only one chromosome pair showed active rDNA sites. In *C. johanna* the active AgNOR bearing pair is 5 for the population AB (Fig. 1B) and 2 for the population CA (Fig. 1D), coinciding with the CH block on the short arm, demonstrating geographic variation for this marker.

Chromosome mapping using 18S rDNA probes, showed multiple hybridization locations that were variable between the two sampled populations of *C. johanna*. Individuals from the AB population showed clusters on only one of the homologs of chromosome pairs 1, 6, 17 and 22 and on both homologs of chromosome pair 5 (Fig. 2A). In comparison, individuals from the CA population only showed clusters on the short arm of chromosome pair 2 and the terminal region of one of the homologs of chromosome pair 16 (Fig. 2B). In *C.* cf*. saxatilis* 18S rDNA probes hybridized around a secondary constriction in the short arm of chromosome pair 1 (Fig. 2C). In *C.* cf*. regani* 18S rDNA probe hybridization was observed along the short arm of chromosome pair 2 (Fig. 2D). In *C.* sp*.* ‘Xingu I’ hybridization occurred in the terminal region of one acrocentric chromosome pair (Fig. 2E). In this case, it was impossible to determine which chromosome pair because of the great similarity in size and acrocentric morphology of so many chromosome pairs in the karyotype.

In all four species, fluorescent *in situ* hybridization (FISH) with telomeric probes (TTAGGG) showed hybridization locations in the distal regions of all chromosomes (Fig. 2). In *C. johanna* variable numbers of hybridization location clusters were observed that were syntenic with 18S rDNA locations. There were six such clusters in individuals from the AB population and three clusters in individuals from the CA population (Fig. 2A,B). In both cases Fiber*-*FISH analysis revealed that telomeric and 18S rDNA sequences are associated and arranged in an intercalated manner (Fig. 2A). Interstitial telomeric sequences (ITS) were found in *C.* cf*. saxatilis*, located in the pericentromeric region of chromosome pair 1 (Fig. 2C).

In all species the 5S rDNA probes hybridized in an interstitial region of one of the acrocentric chromosome pairs (Fig. 2F).

## DISCUSSION

### Karyoevolution in *Crenicichla*

The conserved diploid number of 48 chromosomes in the four species of *Crenicichla* analyzed here is common to 75% of species in the subfamily Cichlinae (Schneider et al., 2013; Perazzo et al., 2011).

However, the data produced here shows for the first time a completely different karyotype in the species *C.* sp. ‘Xingu I’ with 48 acrocentric chromosomes (FN=48, see Fig. 1I), differing from all other *Crenicichla* species analyzed to date that have some metacentric or submetacentric chromosomes. This pattern (2n=48 and FN=48) is similar to the proposed ancestral karyotype of Feldberg et al. (2003) where a comparison of cytogenetic data from many species of Cichlidae and phylogenetic data (Farias et al., 2000) were used to propose the idea that Neotropical cichlids, including *Crenicichla*, show a strong tendency for chromosomal rearrangement as a result of pericentric inversions. Considering that hypothesis, we explain the origin of the *C.* sp. ‘Xingu I’ karyotype in one of two ways: (1) the species maintained the ancestral conserved karyotype; or (2) the species suffered new inversion events to develop a derived karyotype that is similar to the ancestral form.

The karyotype described here for *C.* cf*. saxatilis* (8M/SM and 40ST/A) is different to that described for *C. saxatilis* by Oyhenart-Pereira et al. (1975) (6M/SM and 44ST/A), revealing inter-population or interspecific (in case they represent undescribed cryptic species) variation, with the relative increase of a pair of M/SM chromosomes and an equivalent reduction in the number of acrocentric pairs in the populations studied here. This information, associated with the presence of ITS in the pericentromeric region of chromosome pair 1 (M/SM) (see Fig. 2C), suggests that the population studied here shows a recent pericentric inversion in an acrocentric pair to form chromosome pair 1. The pericentric ITS represents the remains from the inversion process as suggested for the snake *Corallus hortulanos* (Viana et al., 2016), and in the rodent genera *Microtus* (Rovatsos et al., 2011) and *Phodopus* (Paço et al., 2012).

The presence of 5S rDNA at the interstitial region of an acrocentric chromosome pair in all four species represents a conserved characteristic in the Cichlidae (Nakajima et al., 2012). However, previous research described variation in the distribution of this marker in *Crenicichla lepidota*, with the presence of 5S rDNA in the interstitial region of two acrocentric chromosome pairs instead of one pair (Perazzo et al., 2011). Studies in various species of fishes have shown that 5S rDNA hybridization can present variation in number, structure and origin of marked locations (Martins et al., 2002; Nakajima et al., 2012; Barros et al., 2017), demonstrating a difference in microstructural organization of the karyotype in the genus, despite the conserved diploid number.

### Association of heterochromatin and repetitive DNA in *C. johanna*

The distribution of CH in pericentromeric and terminal regions of chromosomes is common in species of *Crenicichla* (Mizoguchi et al., 2007; Molina et al., 2014; Pires et al., 2015). The heterochromatinization of parts of the genome can be characterized as a repression of recombinant processes, protecting the genomic integrity of the organism (Grewal and Jia, 2007). As such, the heterochromatinization of the whole short arm of chromosome pair 2 in the CA population of *C. johanna* (Fig. 1D) may be the result of the amplification of CH (Margarido and Galetti, 2000) in the region that contains co-localized telomeric sequences (Fig. 2B). The CH may help stabilize these sites where the syntenic blocks of 18S rDNA would otherwise result in instability with frequent recombination (Ocalewicz, 2013). The size heteromorphism observed between homologues of this chromosome pair may be associated with simple translocations or unequal crossing-over (Szostak and Wu, 1980).

The variability of the distribution of 18S rDNA between populations of *C. johanna*, marking six chromosomes in the AB population and three in the CA population (Fig. 2A,B), may be explained by: (1) ectopic recombination, resulting from the physical approximation of chromosomes during meiotic interphase or prophase, when the marked chromosomes could present the Rab1 or bouquê configurations, respectively, as described for the scorpion *Tityus obscurus* (Almeida et al., 2017); (2) translocations, considering that heterochromatic regions rich in rDNA are also considered hotspots for chromosome breaks (Rousselet et al., 2000); or (3) transposition of active transposable elements (Symonová et al., 2013)

**Fig. 3.**
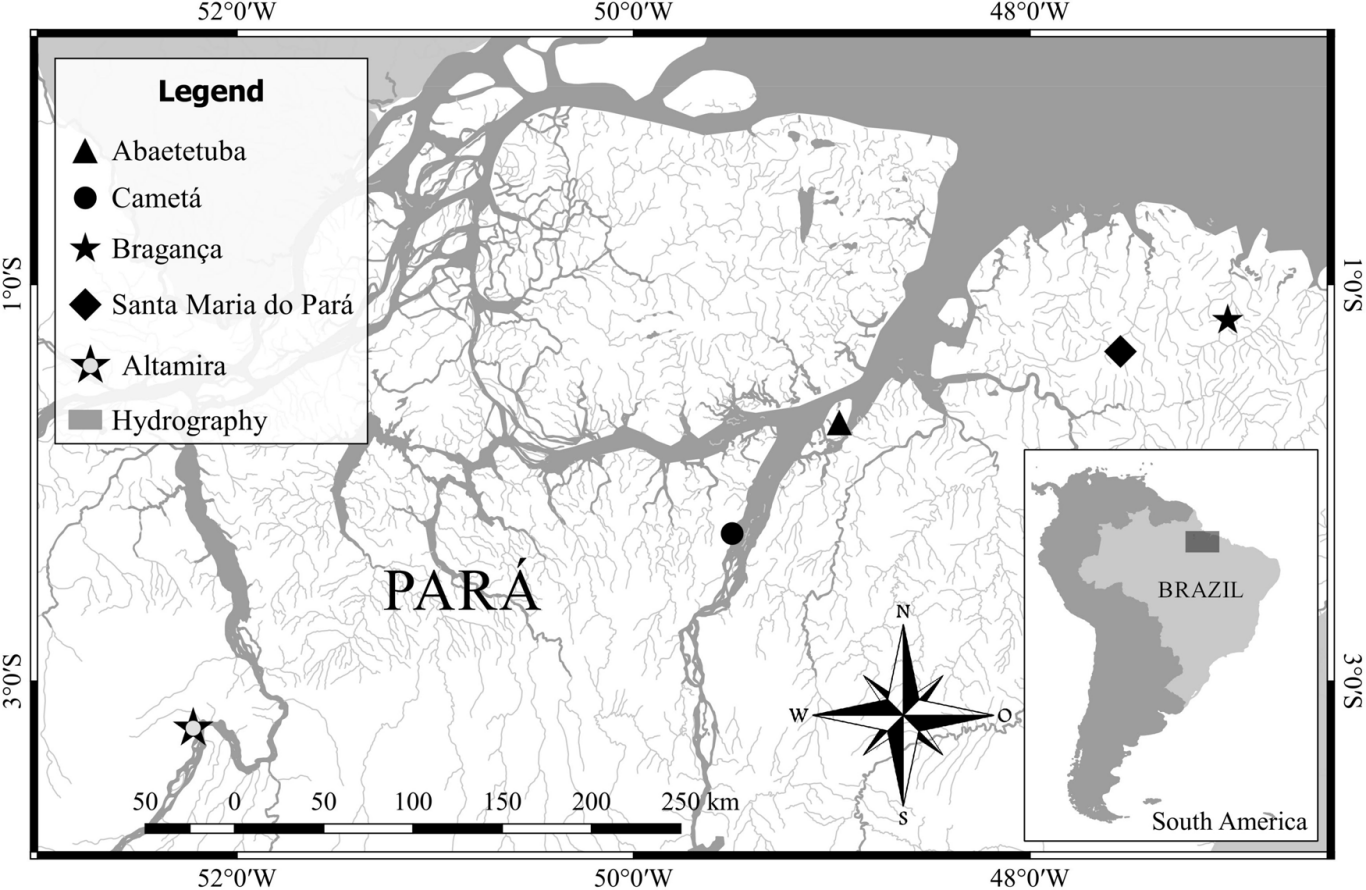
**Map indicating sample collection locations of *Crenicichla* analyzed cytogenetically in this study.**

In addition to the great variability in 18S rDNA sites between the two populations of *C. johanna*, the active NOR bearing chromosome pair differs between populations (Figs 2B,D and 3A,B). Furthermore, the results obtained here are distinct from those of Benzaquem et al. (2008) for a population of *C. johanna* from the Catalão river in the state of Amazonas, Brazil, where the active NOR is on the short arm of chromosome pair 24. This difference may be the result of competition between nucleolar chromosomes with varying capacities for synthesis or organization of small and large nucleoli (Ramirez and Sinclair, 1975), demonstrating that the active 18S rDNA site can vary between populations of *C. johanna*. Alternatively silencing of rDNA sites through the action of other repetitive DNA may occur as observed with As51 satellite DNA (Vicari et al., 2008).

The association of telomeric and 18S rDNA visualized by Fibre-FISH demonstrated the occurrence of various ITSs intercalated with NOR sequences, as proposed for *Anguilla anguilla* and *Anguilla rostrata* (Salvadori et al., 1995). The association of telomeric and rDNA sequences has been observed in plants (Souza et al., 2016) and other fishes (Ashley and Ward, 1993). Although the origin and genomic function of this syntenic association is unknown, some authors suggest that it is a hotspot for chromosomal breaks, increasing the chances of chromosomal rearrangements (Ashley and Ward, 1993; Bolzán, 2011; 2017). It is known that 45S rDNA sequences can generate instability in plants and animals because secondary constrictions that develop during chromosome condensation include fragile sites, promoting chromosome breaks (Huang et al., 2008; Cazaux et al., 2011)

By associating the pattern of ITSs in metacentric pairs described in this study with the karyotype evolution model for Neotropical cichlids of Feldberg et al. (2003), we propose that the association of telomeric and 18S rDNA sequences is strongly related to chromosomal break points that are important for genomic re-patterning in *Crenicichla*.

## MATERIAL AND METHODS

Data describing the samples used in this study and their origin are described in Table 2 and the collection localities indicated in Fig. 3. All samples were deposited in the collection of the Laboratório de Citogenética, Federal University of Pará, Brazil. The taxonomic identification of samples was based on Kullander and Nijssem (1989), Kullander (2003), Ito and Py-Daniel (2015) and Varella and Ito (2017), or, in the case of *C.* sp. ‘Xingu I’, using the commercial trade name for the species which has not yet been formally described (Avila, 2018). When species match original descriptions but come from a single location within a generally wide species distribution, and where new species have been described for populations from some part of the distribution of that original widespread species, we use the conservative identification ‘cf.’ before the specific epithet. Samples were collected under license (SISBIO 13248) and all work performed in accordance with ethical approval by the Federal University of Pará Committee for the Ethical Use of Animals (CEUA 68-2015).

**Table 2.**
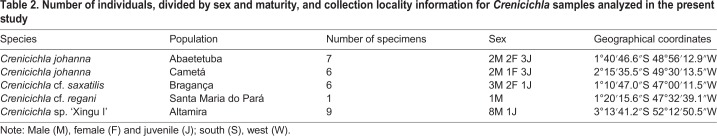
**Number of individuals, divided by sex and maturity, and collection locality information for *Crenicichla* samples analyzed in the present study**

Chromosome preparations were obtained following Bertollo et al. (2015) and classified following Levan et al. (1964). The distribution of CH and nucleolar organizing regions (NORs) were determined following Sumner (1972) and Howell and Black (1980), respectively.

FISH probes for 18S and 5S rDNA were amplified by polymerase chain reaction (PCR) using genomic DNA from *C. johanna*, and the following primer pairs: 18S rDNA (18SF-5′ CCG CTT TGG TGA CTC TTG AT and 18SR-5′ CCG AGG ACC TCA CTA AAC CA) of Gross et al. (2010); and 5S rDNA (5SF-5′ GCC ACA CCA CCC CTG AAC AC and 5SR-5′ GCC TAC GAC ACC TGG TAT TC) of Suarez et al. (2017). In each PCR reaction were used: 100 ng/μl genomic DNA, 2,5 μl of 10× reaction buffer, 0.75 MgCl, 2,0 μl of DNTP mix (2 mM), 1 μl of each primer (10 mM), 1U of enzyme Taq Polymerase (Invitrogen) and 16.5 μl of pure water. The thermal conditions were: a cycle of 94°C for 5 min; 35 cycles composed of 94°C for 1 min, 57–58°C for 1 min and 72°C for 2 min; 1 cycle of 72°C for 10 min; hold at 4°C. Telomeric sequences were amplified by PCR using complementary primers (TTAGGG)n and (CCCTAA)n, without the use of template DNA, following Ijdo et al. (1991).

FISH was performed following Pinkel et al. (1986). The hybridization solution comprised of 2 µl of probe, formamide (50%), 2SSC and dextran sulphate, denatured at 70°C. Denaturation of chromosomal DNA was performed in 70% formamide at 65°C. Hybridization occurred at 37°C, overnight. Probes were detected using avidin-CY3 or anti-digoxigenin-FITC. Chromosomes were counterstained with DAPI containing anti-fading Vectashield.

Fiber-FISH was performed following Barros et al. (2011). Cytologically prepared slides were washed in 1× PBS for 5 min. Chromatin fibers were extended by brushing with 0.15M NaOH diluted in 30% ethanol. Subsequently, 500 μl of ethanol was added directly to the slides, and the material was dehydrated using a battery of ethanol prior to analyses by FISH.

Slides were analyzed on an Olympus BX41 microscope and photographed using a Canon Powershot A95 camera. FISH images were captured using a CCD AxioCam MRm (Nikon) camera coupled to an Epifluorescence Nikon H550S (Nikon) microscope and using the program Nis-Elements (Nikon). Image editing to adjust brightness, contrast and mount karyotypes was performed in Adobe Photoshop CS5.

For all cytogenetic preparations a minimum of ten metaphase cells were analyzed in order to confirm which fluorescence markings represent true hybridization sites and which represent background noise. Examples of some of the metaphase preparations analyzed are available in the supplementary material (Figs S1–S14).

## Supplementary Material

Supplementary information
