# Supplementary material for: Karyoevolution of Crenicichla heckel 1840 (Cichlidae, Perciformes): a process mediated by inversions
Source: Biol Open. 2019 Apr 29;8(5):bio041699. doi: 10.1242/bio.041699 (PMC6550074; doi:10.1242/bio.041699)
Supplement: Supplementary information [file biolopen-8-041699-s1.pdf]

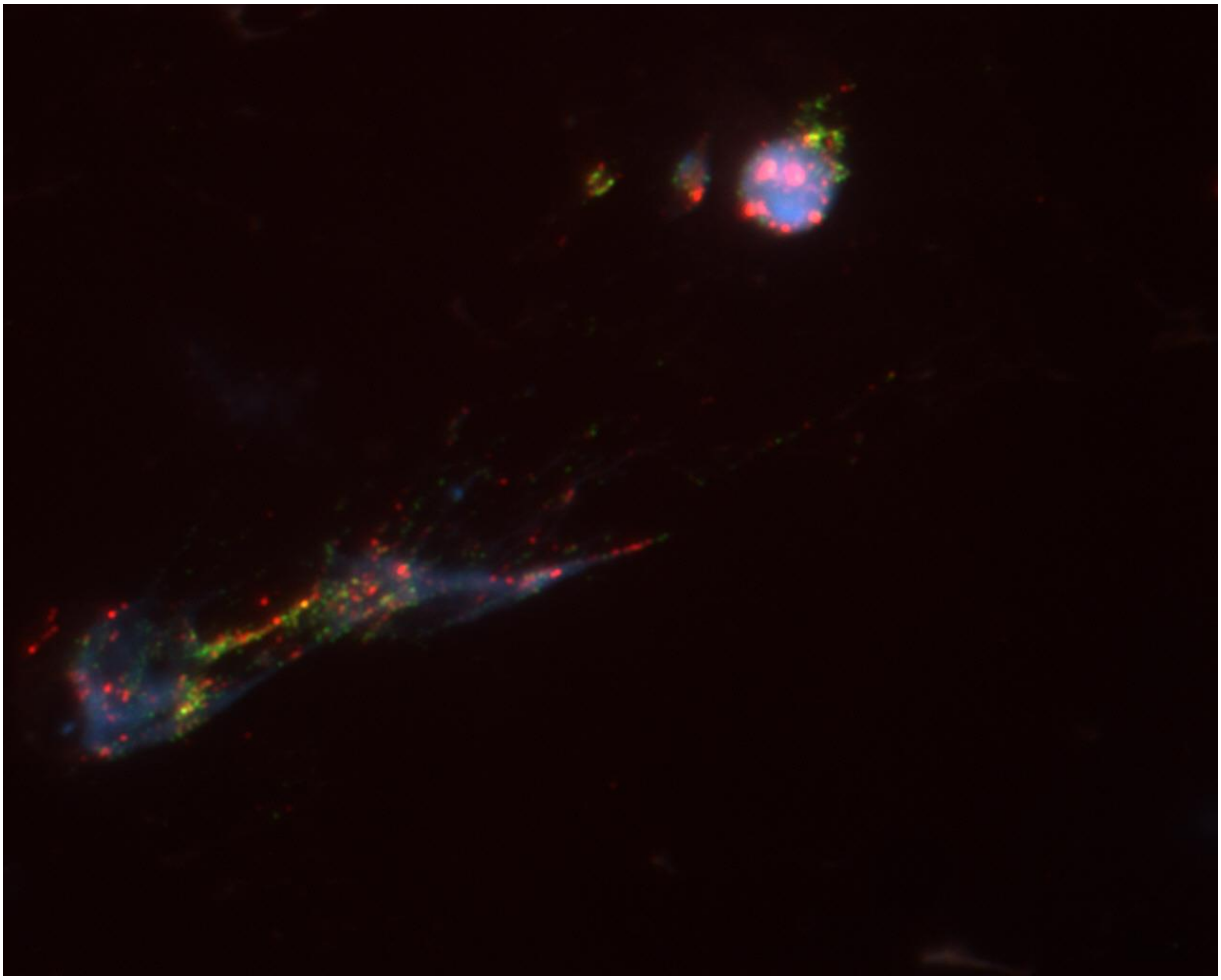

Figure S1. Unedited image of cytogenetic preparation of *C. johanna* - Abaetetuba - fiber FISH - rDNA 18S FITC - Telomere CY3

Biology Open • Supplementary information

Biology Open (2019): doi:10.1242/bio.041699: Supplementary information

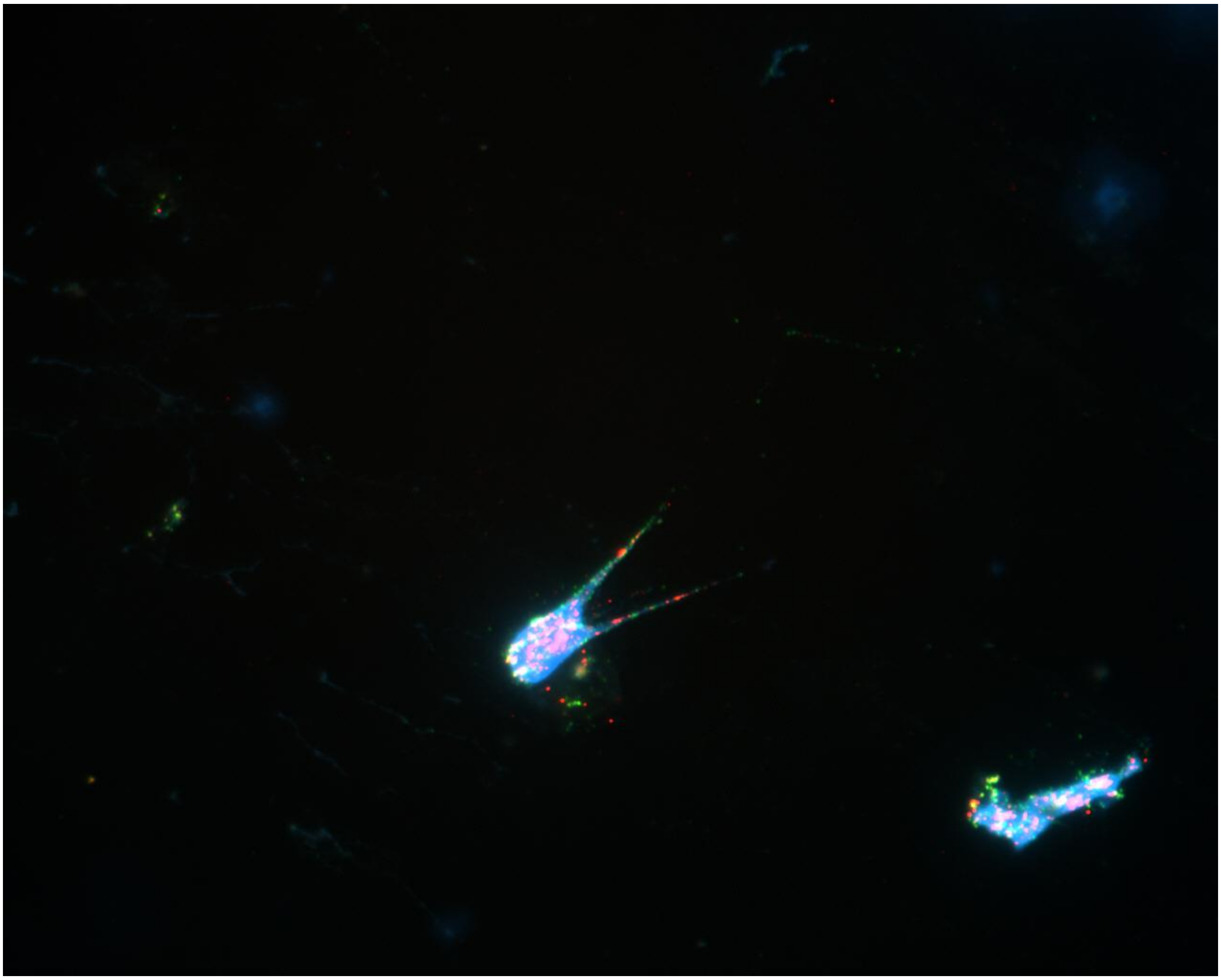

Figure S2. Unedited image of cytogenetic preparation of *C. johanna* - Abaetetuba - fiber FISH - rDNA 18S FITC - Telomere CY3 - 2

Biology Open • Supplementary information

Biology Open (2019): doi:10.1242/bio.041699: Supplementary information

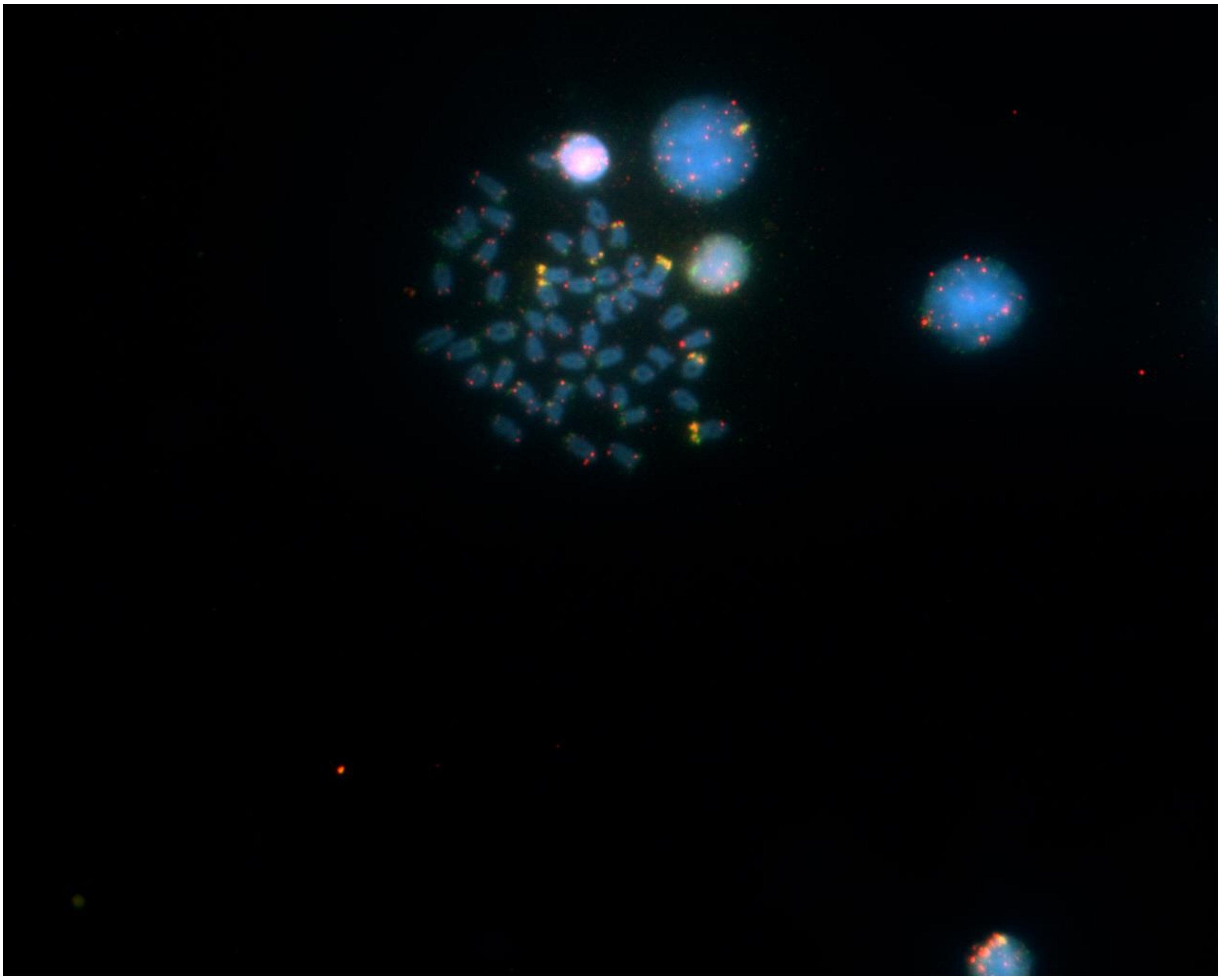

Figure S3. Unedited image of cytogenetic preparation of *C. johanna* - Abaetetuba - rDNA 18S FITC - Telomere CY3

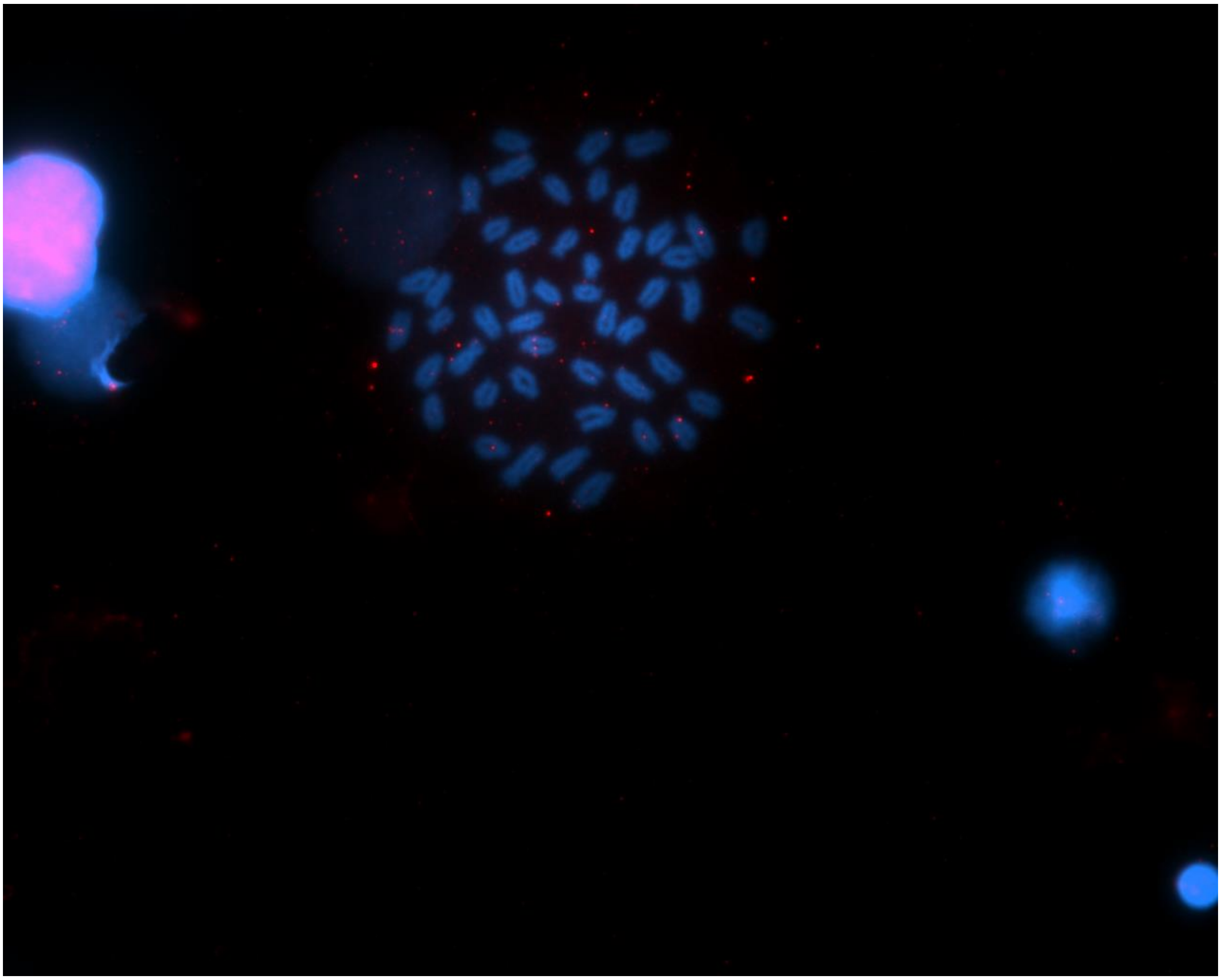

Figure S4. Unedited image of cytogenetic preparation of *C. johanna* - Abaetetuba - rDNA 5S - CY3

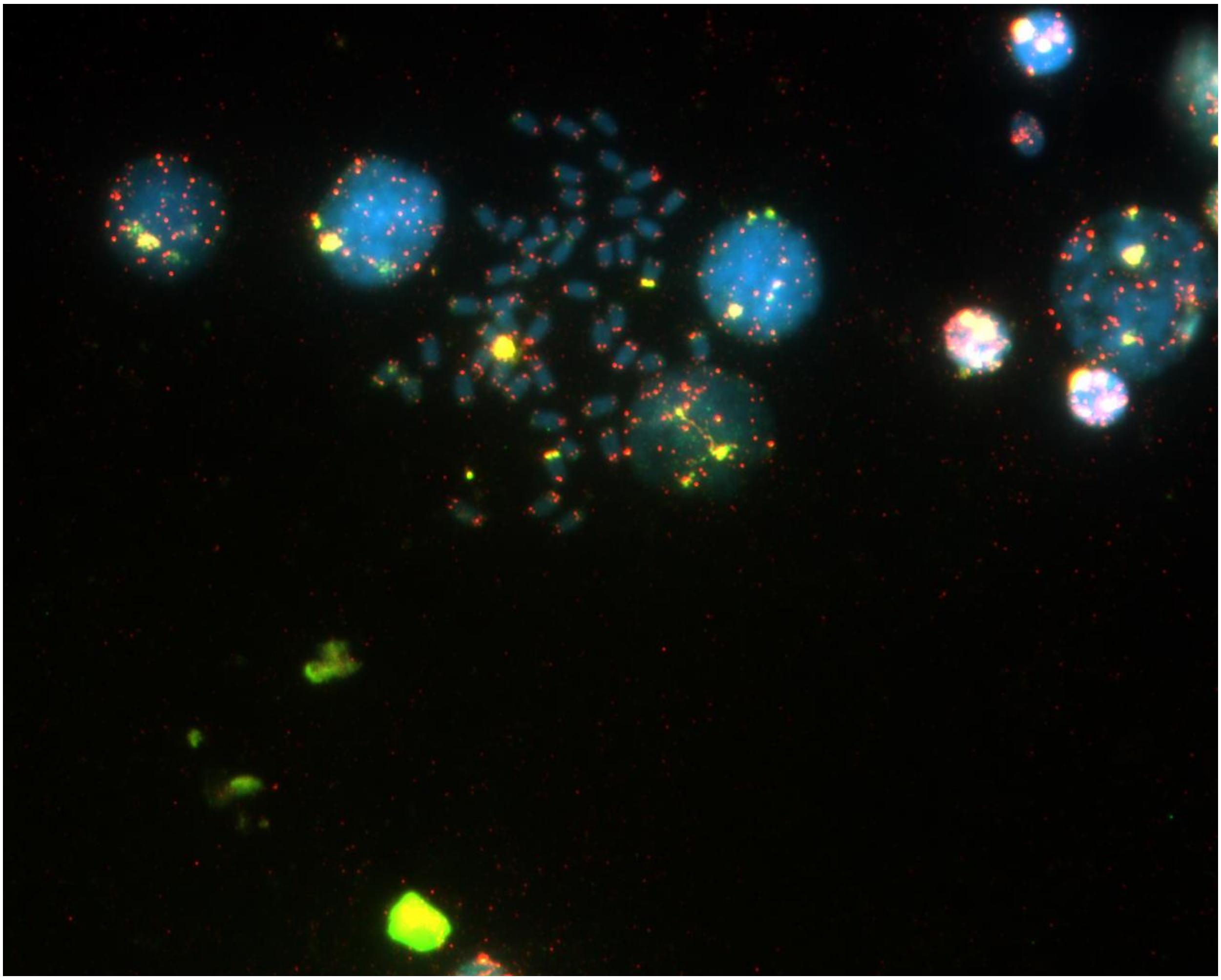

Figure S5. Unedited image of cytogenetic preparation of *C. johanna* - Cametá - rDNA 18S FITC - Telomere CY3

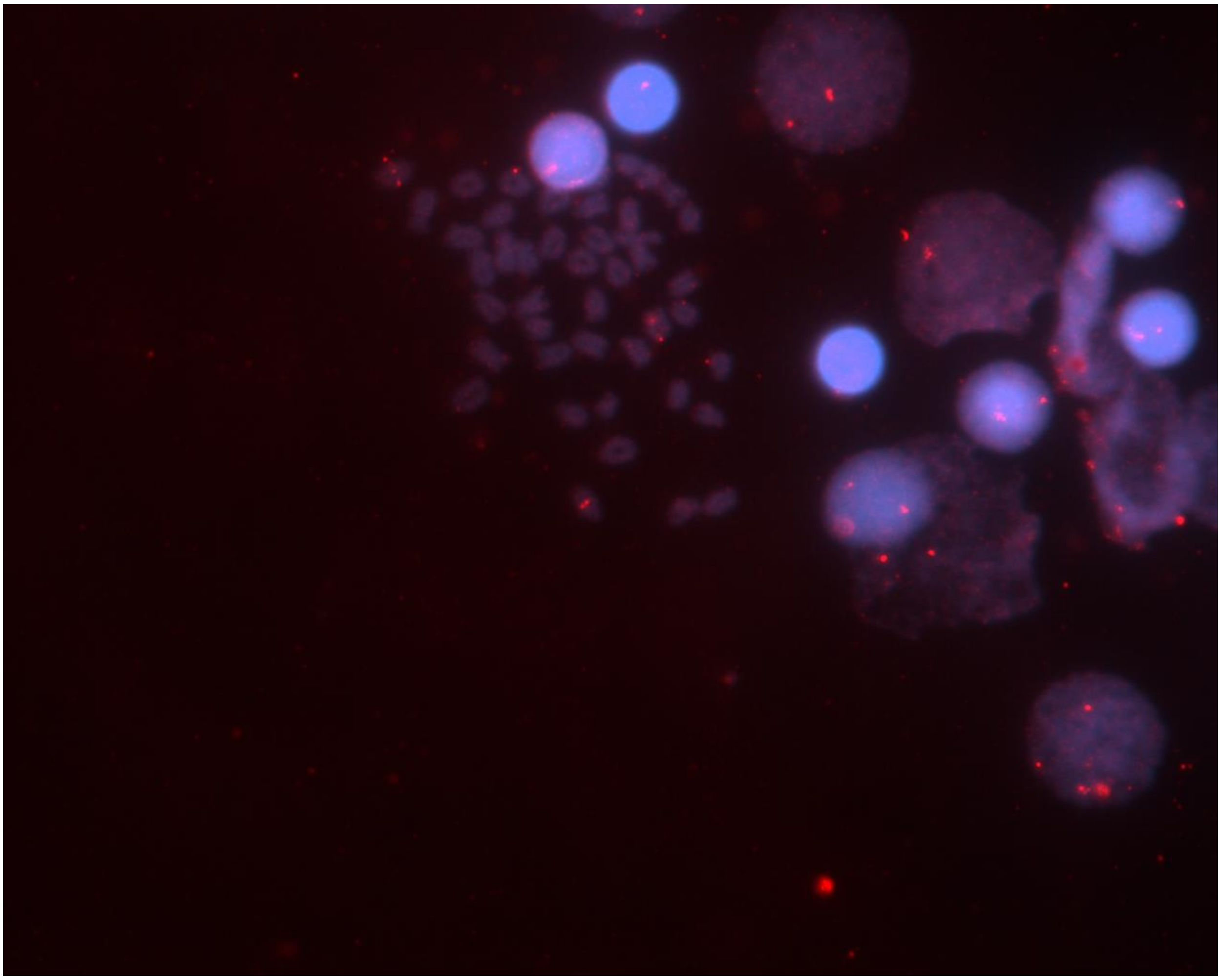

Figure S6. Unedited image of cytogenetic preparation of *C. johanna* - Cametá - rDNA 5S - CY3

Biology Open • Supplementary information

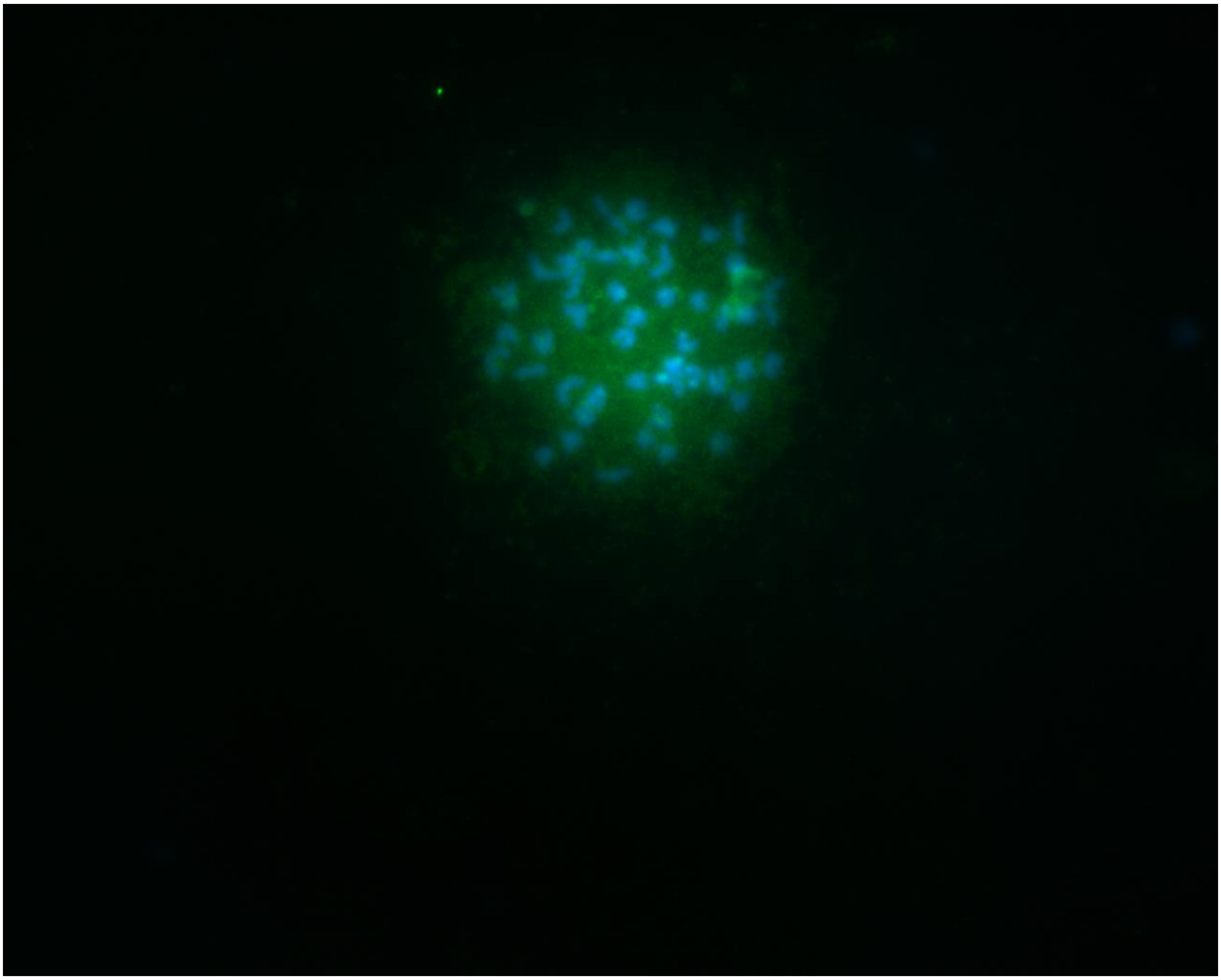

Figure S7. Unedited image of cytogenetic preparation of *C. regani* - rDNA 18S FITC

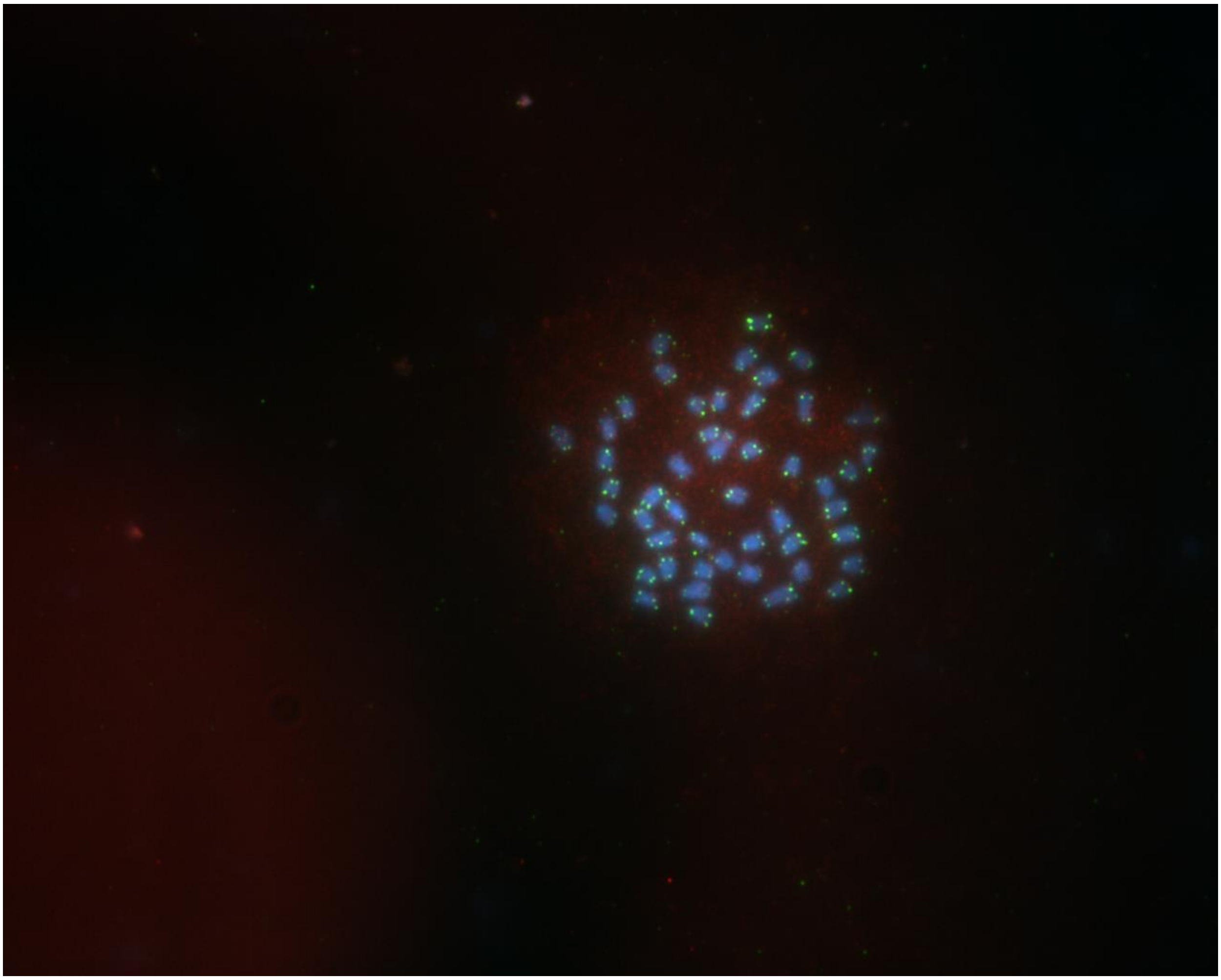

Figure S8. Unedited image of cytogenetic preparation of *C. regani* - Telomere FITC

Biology Open • Supplementary information

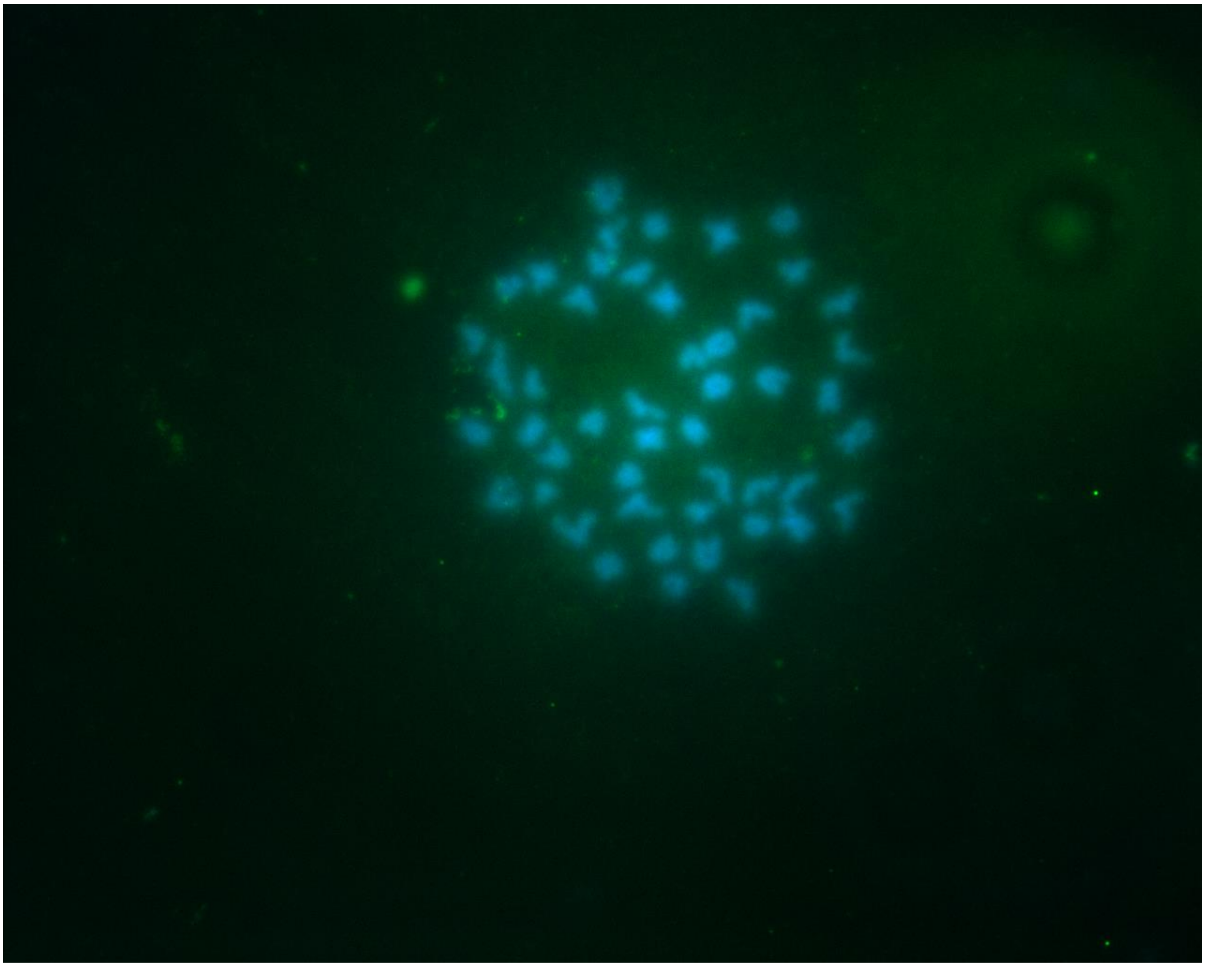

Figure S9. Unedited image of cytogenetic preparation of *C. regani* - rDNA 5S - FITC

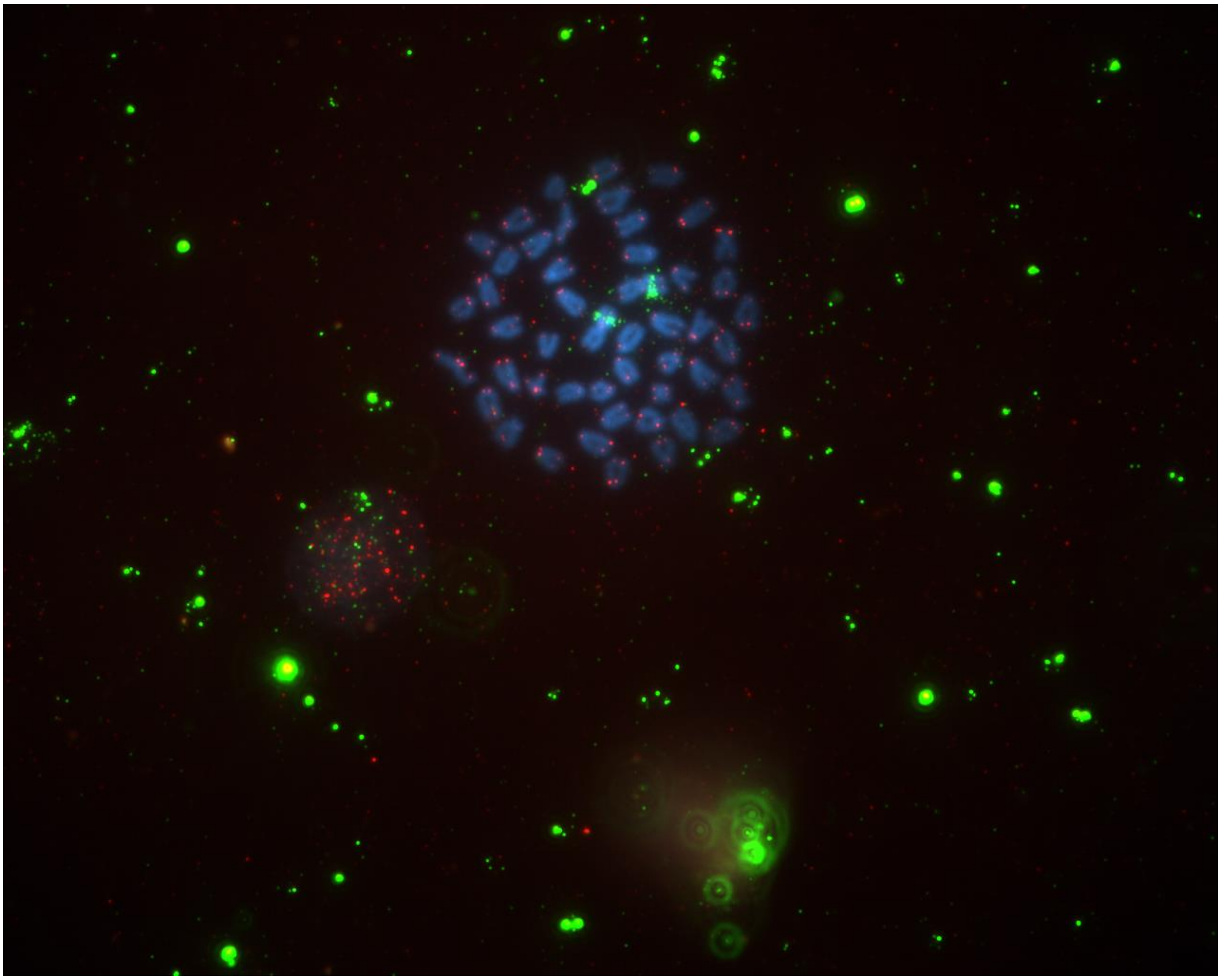

Figure S10. Unedited image of cytogenetic preparation of *C. saxatilis* - rDNA 18S FITC - Telomere CY3

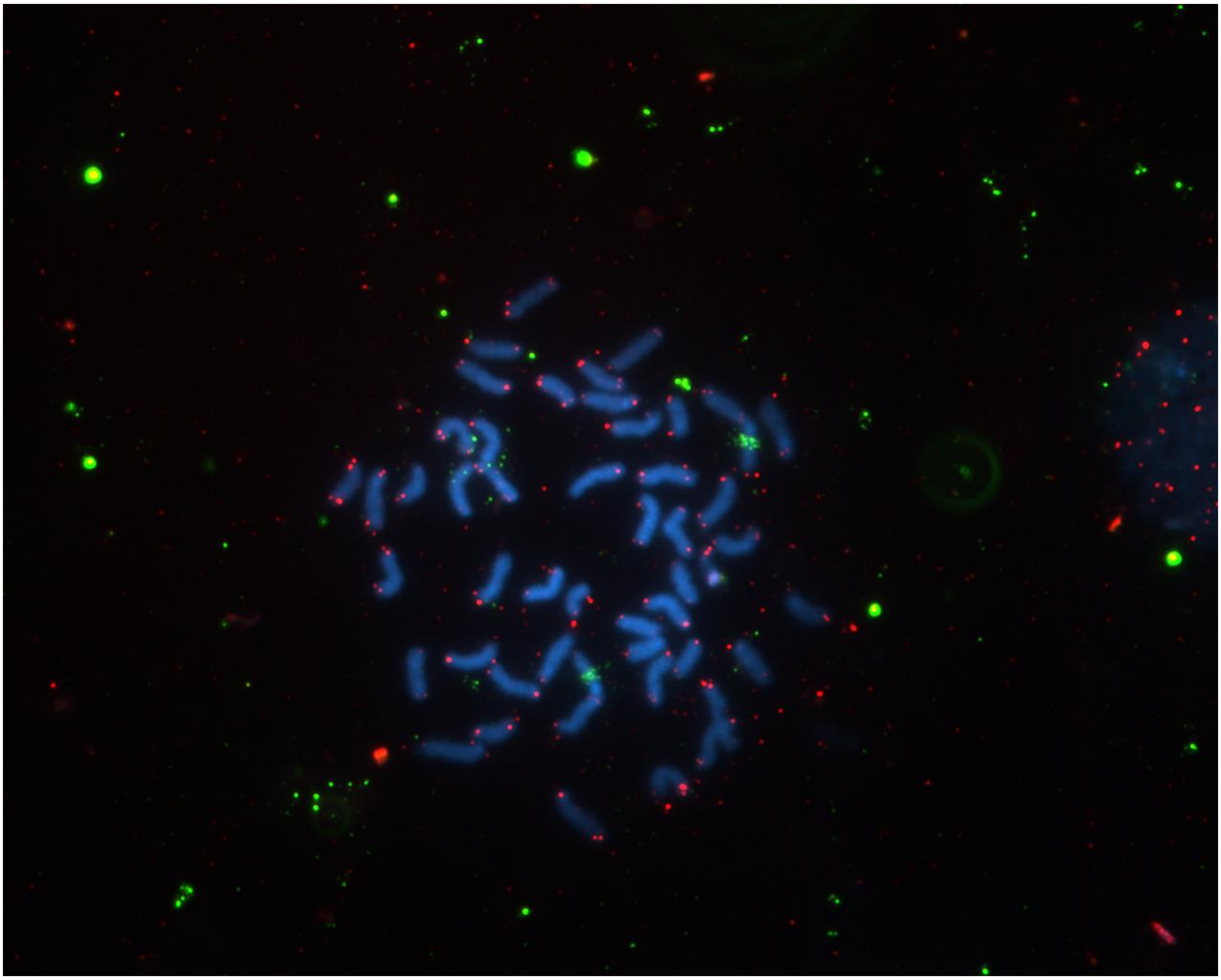

Figure S11. Unedited image of cytogenetic preparation of *C. saxatilis* - rDNA 18S FITC - Telomere CY3 - 2

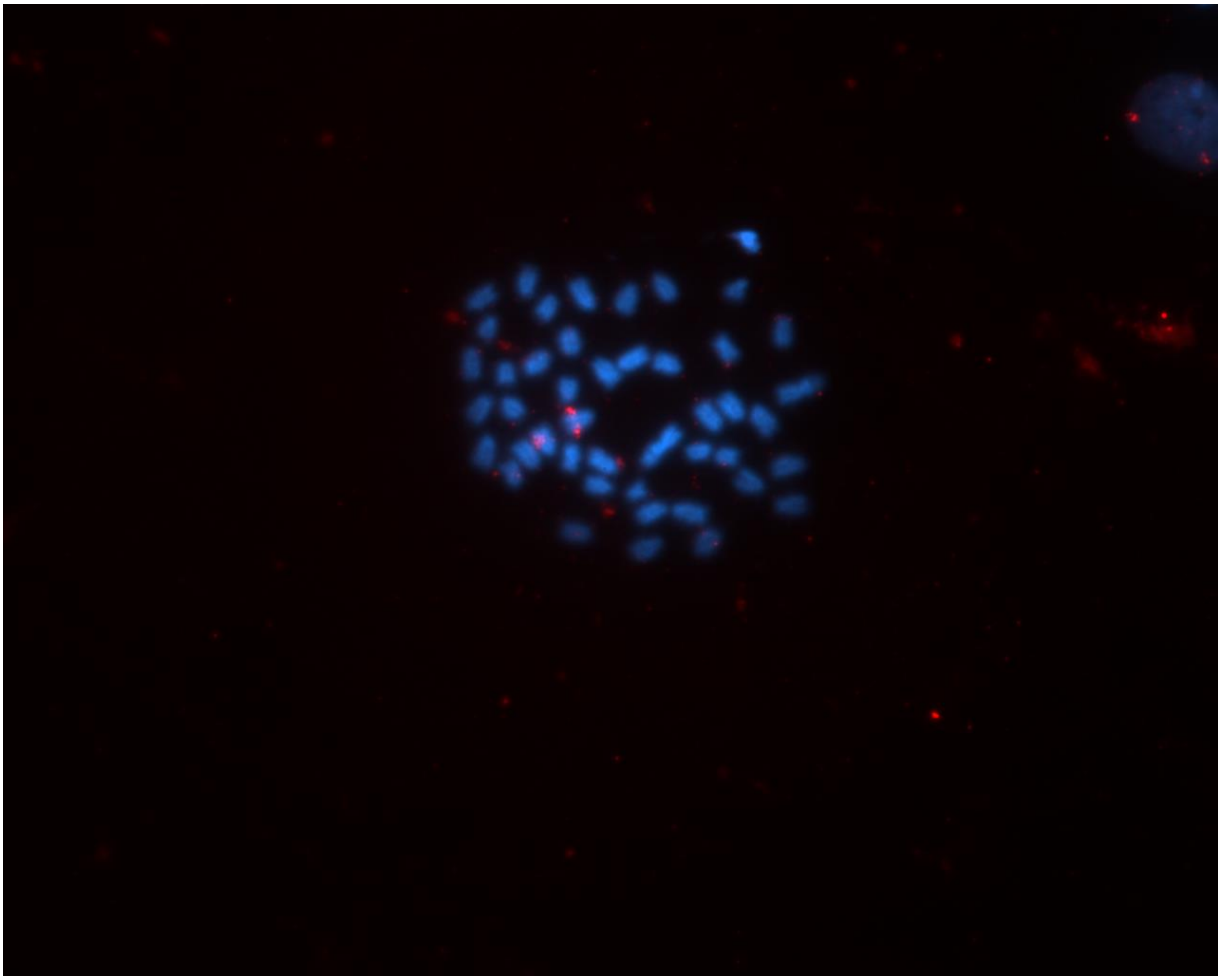

Figure S12. Unedited image of cytogenetic preparation of *C. saxatilis* - rDNA 5S - CY3

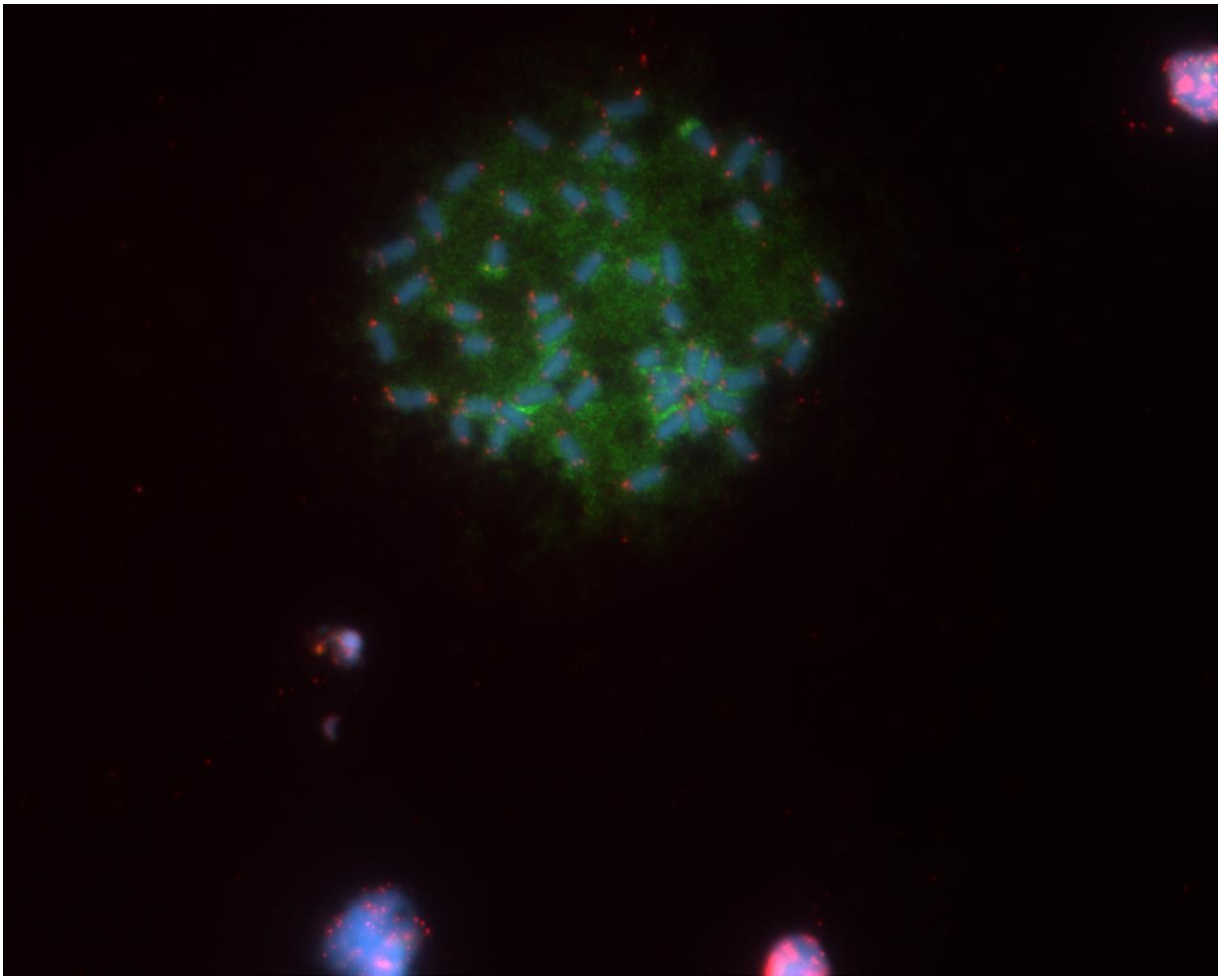

Figure S13. Unedited image of cytogenetic preparation of *Crenicichla* sp. “Xingu I” - rDNA 18S FITC - Telomere CY3

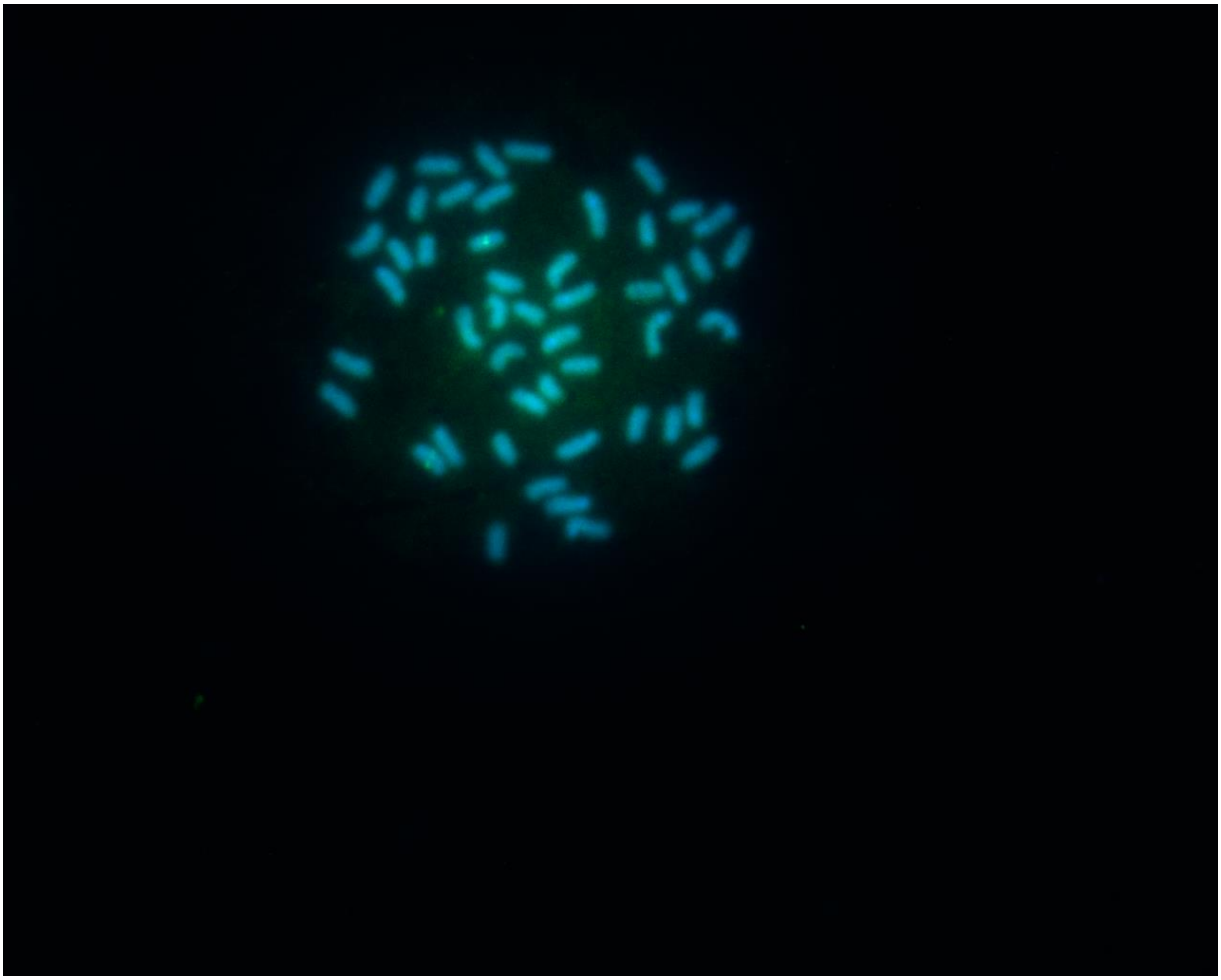

Figure S14. Unedited image of cytogenetic preparation of *Crenicichla* sp. "Xingu I" - rDNA 5S - FITC
